# Distinct impacts of high intensity caregiving on caregivers’ mental health and continuation of caregiving

**DOI:** 10.1186/s13561-017-0151-9

**Published:** 2017-04-07

**Authors:** Narimasa Kumagai

**Affiliations:** Faculty of Economics, Kindai University, 3-4-1 Kowakae, Higashiosaka, Osaka 577-8502 Japan

**Keywords:** Control function approach, High-intensity caregiving, Informal caregiver, Japan, Mental health, State dependence

## Abstract

Although high-intensity caregiving has been found to be associated with a greater prevalence of mental health problems, little is known about the specifics of this relationship. This study clarified the burden of informal caregivers quantitatively and provided policy implications for long-term care policies in countries with aging populations. Using data collected from a nationwide five-wave panel survey in Japan, I examined two causal relationships: (1) high-intensity caregiving and mental health of informal caregivers, and (2) high-intensity caregiving and continuation of caregiving. Considering the heterogeneity in high-intensity caregiving among informal caregivers, control function model which allows for heterogeneous treatment effects was used.

This study uncovered three major findings. First, hours of caregiving was found to influence the continuation of high-intensity caregiving among non-working informal caregivers and irregular employees. Specifically, caregivers who experienced high-intensity caregiving (20–40 h) tended to continue with it to a greater degree than did caregivers who experienced ultra-high-intensity caregiving (40 h or more). Second, high-intensity caregiving was associated with worse mental health among non-working caregivers, but did not have any effect on the mental health of irregular employees. The control function model revealed that caregivers engaging in high-intensity caregiving who were moderately mentally healthy in the past tended to have serious mental illness currently. Third, non-working caregivers did not tend to continue high-intensity caregiving for more than three years, regardless of co-residential caregiving. This is because current high-intensity caregiving was not associated with the continuation of caregiving when I included high-intensity caregiving provided during the previous period in the regression. Overall, I noted distinct impacts of high-intensity caregiving on the mental health of informal caregivers and that such caregiving is persistent among non-working caregivers who experienced it for at least a year. Supporting non-working intensive caregivers as a public health issue should be considered a priority.

## Background

Co-residential informal caregiving leads to increased stress and lowered psychological health. The intensity of caregiving in co-residential situations appears to be much greater than in extra-residential ones, and this high-intensity caregiving (i.e., 20 or more h per week) leads to especially poor health among family caregivers. “Intensive carers,” defined as those who provide more than 20 h of care per week, are more likely to stop working and to have worse mental health outcomes as a result of their caregiving responsibilities [1]. Drawing on British Household Panel Survey data (1991–2000), Hirst showed that individuals who provided high-intensity caregiving had double the risk of psychological distress as did non-caregivers [2].^1^ Notably, this effect was greater in women. Colombo et al. argued that high-intensity caregiving is associated with a higher risk of poverty [1].^2^ Despite these findings, relatively little is known about the precise impact of high-intensity caregiving on mental health. To address this, I focused on two causal relationships among informal caregivers: between high-intensity caregiving and mental health, and between high-intensity caregiving and continuation of caregiving. The latter relationship was examined because a longer duration of high-intensity care appears to exhaust caregivers to a greater degree than does a shorter duration. As such, I wanted to clarify how caregivers’ informal caregiving changes with psychological distress during care provision.

In 2006, Japan revised its social long-term care insurance (LTCI) entitlement for mildly disabled older people into a “prevention system,” which aims to help those eligible for support to better maintain their independence.^3^ Such approaches can be combined with more adequate support strategies for family caregivers [3]. Colombo et al. suggested that supporting family caregivers is effectively a win-win solution, because it involves far less public expenditure for a given amount of care [1]. Indeed, the support of family caregivers is an important public health issue, both in Japan and worldwide.^4^ Thus, I aimed to determine whether respite care is useful for supporting the mental health of informal caregivers engaged in high-intensity caregiving. Some previous studies have demonstrated that respite care has a positive effect on caregivers.^5^ It is possible that the influence may be greater for caregivers engaged in high-intensity care, while day care appears to be more effective for carers in paid employment (i.e., who are engaged in less intensive care) [4]. Furthermore, the use of a short-term stay service funded by the LTCI has demonstrated positive effects on the well-being of family caregivers. This service is perhaps the most efficient, followed by home-helper services [5]. Greater use of day-care and respite short-stay services have indicated that such services might provide traditional female caregivers with temporary relief from their care burden [6].^6^

As stated before, I investigated the longitudinal associations between high-intensity caregiving and caregivers’ mental health, and between high-intensity caregiving and continuation of caregiving in an older adult population. A random-effects probit estimation was employed to reveal the determinants of both caregivers’ mental health and continuation of caregiving. However, it must be noted that caregivers often make adjustments in employment status to facilitate caregiving, such as reducing work hours or quitting work altogether [7]. Therefore, it seemed necessary to classify informal caregivers by their employment status (regular employees, irregular employees, and non-working caregivers). In dividing participants by their employment status, I was aware that I would also be dividing participants by their intensity of informal caregiving. This was supported by the fact that a preliminary analysis indicated a difference in hours of caregiving between various employment status groups, even after controlling for other socioeconomic and demographic characteristics. Considering this heterogeneity in high-intensity caregiving among informal caregivers by employment status, I estimated mental health functions using the control function approach. This approach allows for assessment of heterogeneous treatment effects combined with self-selection of treatment.

This paper is organized as follows. Section 2 provides a literature review of the effects of high-intensity caregiving on caregivers’ health and formal care use. Section 3 outlines the characteristics of the nationally representative sample used in this study and the variables of interest, such as caregivers’ mental health. In Section 4, I describe the empirical methods and report the estimation results. Section 5 contains conclusions.

### Effects of high-intensity caregiving on caregivers’ health and formal care use

Caregivers are at risk of becoming patients themselves. Furthermore, most studies have found that caregiver burden is higher for women than for men. This discrepancy may be partially explained by how traditional gender roles place greater pressure on women to commit to the caregiver role [8].^7^ Additionally, Brouwer et al. reported that disrupted life schedules and caregivers’ health problems were the strongest predictors of subjective burden scores [9].

Objective burden must be considered independently of subjective burden, since caregivers’ mental state can alter their perception of their personal burden, regardless of their actual burden [10]. The objective burden of informal caregiving is typically defined as the amount of time spent on this activity. Brouwer et al. found that a greater time spent on caregiving is related to reduced quality of life and probability of having paid employment in the labor force [9].^8^ Using a population-based sample, Beach et al. found that an increasing intensity of care was associated with increasingly poor (mental) health for caregivers [11]. Houser and Gibson similarly found that the greater the intensity of caregiving, the greater the magnitude of the health effects due to chronic stress [12]. Additionally, caregiving, particularly intensive caregiving, reduced female labor force participation and hours worked [13–15].

In Japan, informal caregivers can purchase formal care services from LTCI, depending on the eligibility levels of care recipients (i.e., their care needs or support level). Individuals who engage in less intensive caregiving might prefer formal care to informal care—for example, workers with caregiving responsibilities, as it may ease the intensity of informal caregiving and allow them to continue their work. Sugawara and Nakamura found that regular workers are more likely to utilize formal care, whereas non-regular workers tend to provide informal care by themselves [16].^9^ Informal care can serve as a substitute for formal long-term care in the sense that elderly parents can receive needed assistance (e.g., in eating or taking a bath) from their children [17].^10^ Indeed, Kikuchi confirmed that informal care by a co-residential caregiver had a negative effect on the use of institutional care services [18].

In the present study, changes in work status during care provision were assumed to occur for exogenous reasons, despite the fact that many informal caregivers make simultaneous decisions about formal care use, informal caregiving, labor participation, and living arrangements. Furthermore, due to the limited data on formal care services, the availability of such services was not taken into account in the present study. Instead, I created a proxy variable of formal care use as a dummy variable of respite care, which took on the value of 1 if hours spent on caregiving exceeded work hours in the past year and work hours exceeded hours spent on caregiving this year. Using this dummy variable, I examined whether respite care was useful for informal caregivers in reducing the negative effects of high-intensity caregiving.

## Methods

### Data

The data used in this study were drawn from five waves of the Longitudinal Survey of Middle-aged and Elderly Persons (LSMEP; 2005–2009) conducted by the Japanese Ministry of Health, Labor and Welfare (MHLW). The LSMEP is a nationally representative sample of the near elderly in Japan (individuals aged 50–59 entered the sample initially). The LSMEP collects information about family situation, health status, and employment status on an annual basis using self-report questionnaires. Samples in the first wave were collected nationwide in November 2005 through a two-stage random sampling procedure.^11^

### Samples

I examined regular employees, irregular employees, and non-working caregivers separately, given their different propensities for providing informal care. I further delineated non-working caregivers by social status.^12^ Individuals who were not working were categorized as workers during a period of family care leave or unemployment, or as otherwise inactive persons (including homemakers and retirees). I defined informal caregivers who were seeking employment as unemployed. Informal caregivers who did not respond to the questions about labor participation and earned income were defined as “non-working caregivers.” Most Japanese companies have a mandatory retirement system—at the time of the 2005 survey, employees were allowed to work until age 60; however, the official employable age was raised from 60 to 65 in 2013.

To explore the effects of high-intensity caregiving on the mental health of informal caregivers, I utilized only those LSMEP respondents who were reported caregivers. To isolate these individuals, I created a respondent-level dataset, and then limited the sample to individuals who had responded to the question on hours of informal caregiving. The subjects who had not filled out this information were excluded. In total, this sample comprised 15,273 subjects, including 3,445 inactive persons (e.g., homemakers, retirees), 784 workers during a period of family care leave, 486 unemployed individuals, 6,978 irregular employees, and 3,580 regular employees. Because poor mental health status might occur due to unemployment, I excluded unemployed caregivers in the regression analysis.

### Main measures

#### Dependent variables

As a measure of objective mental health measures, the Kessler 6 non-specific distress scale (K6) developed by Kessler et al. [19] was used. The K6 is a six-item psychological screening instrument that was included in the LSMEP. The K6 scale asked respondents how frequently they experienced the following six symptoms: “During the past 30 days, about how often did you feel a) nervous, b) hopeless, c) restless or fidgety, d) so depressed that nothing could cheer you up, e) that everything was an effort, and f) worthless?” For each question, participants answered on a 5-point scale where responses of “none of the time,” “a little of the time,” “some of the time,” “most of the time,” or “all of the time” were assigned values of zero, one, two, three, and four, respectively. The responses to the six items were summed to yield a K6 score between 0 and 24, with higher scores indicating a greater tendency towards mental illness. A K6 cut-off point of 13 was established to operationalize the definition of “serious mental distress.” Moderate mental distress was defined as 5 ≤ K6 < 13.^13^ However, as Oshio [20] argued, because the results were not free from potential biases due to their self-reported nature, I created a dichotomous variable of serious mental illness as well as an ordinal variable (serious = 2, moderate = 1, otherwise = 0).

#### Primary explanatory variables

A preliminary analysis revealed that non-working caregivers spent more hours on caregiving per week on average than did working caregivers. Specifically, the proportions of individuals who engaged in high-intensity caregiving (20 or more hours per week) were as follows: regular employees, 14.4%; irregular employees, 20.8%; inactive persons, 27.2%; unemployed, 26.5%; and care leave, 50.3%.^14^

It is important to be aware of the non-proportional relationship between hours of caregiving and caregivers’ perceived burden. Assessing caregivers’ burden with the Zarit Burden Interview, Arai and Ueda confirmed that these two variables are not directly connected [21]. Thus, because longer duration of caregiving does not directly mean that caregivers will have greater burden, simply measuring the presence or absence of high-intensity caregiving might not be an adequate explanatory variable of continuation of caregiving. Therefore, I used three dichotomous variables to measure intensity of caregiving, as follows: (I) 20 or more hours per week = 1, otherwise = 0; (II) more than 20 h and less than 40 h per week = 1, otherwise = 0; and (III) 40 or more hours per week = 1, otherwise = 0. Note that (I) includes (II) and (III).

The LSMEP included items relating to the family member(s) for whom respondents provided care (i.e., father, mother, father-in-law, mother-in-law, and others) at the time of the study. I constructed four binary variables indicating informal care provision for the four family member types (i.e., father, mother, father-in-law, mother-in-law). These dummy variables are the main explanatory variables of interest because a preliminary analysis revealed that the proportion of co-residential caregivers engaged in high-intensity caregiving was relatively higher for care recipient who was a mother or mother-in-law.^15^ Furthermore, because co-residential caregivers might commit to more hours of informal care than extra-residential caregivers, and co-residence may reflect having care recipients with higher care needs, co-residential care is often used as a proxy for more intensive care [22, 23].^16^ Co-residential adult children have traditionally been the main caregivers in Japan; for example, in 2010, 64% of caregivers for care recipients in the home were co-residential family members.^17^ Caregiving for a resident parent is associated with depressive symptoms and sleeping problems; indeed, even just having a parent in need of care increases the likelihood of depression [24].

The effect of respite care was measured by using a time-averaged proxy variable of formal care use. I created this proxy variable to use as a dummy variable of respite care, which took on the value of 1 if hours spent on caregiving exceeded work hours in the previous year and work hours exceeded hours spent on caregiving this year; otherwise it took on the value of 0. 18

### Empirical analysis

#### Dynamic random-effects probit model and control function (CF) approach

The specifications of the high-intensity caregiving function dictate that the response probability of a positive outcome depends on unobserved effects and past experience. It is important to consider unobserved heterogeneity because ignoring it can lead to overestimation of the degree of state dependence. The random-effects probit model specification allows for unobserved heterogeneity but it treats the initial conditions as exogenous. Estimating a standard uncorrelated random-effects probit model implicitly assumes zero correlation between the unobserved effect and set of explanatory variables.^19^ Treatment of the initial conditions in a dynamic random-effects probit (DREP) model is crucial, since misspecification will result in an inflated parameter of the lagged dependent variable term. Additionally, ignoring the initial conditions problem yields inconsistent estimates [25, 26]. Kumagai and Ogura is a study which overcomes the initial conditions problem [27]. Using the procedure described in [26], they estimated DREP models and revealed that the degree of dependence between previous health stock and current health stock exhibited moderate persistence.^20^

I estimate the DREP models of high-intensity caregiving function in this study. The core econometric specification of this function is as follows:

1 $$ H{I}_t = f\left({\beta}_C{C}_t + {\beta}_{HI} H{I}_{\mathrm{t}-1} + H C{\hbox{'}}_t{\beta}_{HC} + D{\hbox{'}}_t{\beta}_D + {\beta}_S{S}_t + {\beta}_W{W}_t + X{\hbox{'}}_t{\beta}_X\right) $$

where *HI* is a measure of high-intensity caregiving, *C* a measure of co-residential caregiving, *HC′* a vector of the health status of caregivers, *D′* a vector of demographic variables, *S* the presence of social relationships (having friends/acquaintances), *W* the wage rate of caregivers or the relative resources of non-working caregivers, and *X′* a vector of other socioeconomic variables. *β*_*C*_, *β*_*HI*_, *β*_*HC*_, *β*_*D*_, *β*_*S*_, *β*_*W*_, and *β*_*X*_ are the coefficients to be estimated. The subscript *t* indexes time periods. In this analysis, the function *f* is the probit function when *HI* is dichotomous. All models included the following covariates: co-residential caregiving; informal care provision for each of the four types of family members; age; marital status (married, divorced, or widowed, with never married as the comparison group); current employment; educational attainment (junior-high education, some post-secondary education, university degree, and graduate degree, with high school completion as the comparison group).

As mentioned above, the subsamples by employment status had differing intensities of informal caregiving. Thus, I am treating the heterogeneity as individual-specific in estimating the effect of explanatory variables on the outcomes of interest.^21^ I examined two causal relationships: (1) high-intensity caregiving and mental health of informal caregivers, and (2) high-intensity caregiving and continuation of caregiving. Considering the heterogeneity in high-intensity caregiving among informal caregivers, a method that allows for heterogeneous treatment effects combined with self-selection into treatment is necessary for appropriate analysis of these relationships. Thus, I estimated the following control function model.

When unobservables, *u*_*i,t*_ and *ε*_*i,t*_, are assumed to be linearly related to *v*_*i,t*_ 22 and all unobservables are assumed to be independent of *z*_*i,t*_ (i.e., covariates used for modeling the outcome), then *v*_*i,t*_ will have a zero mean. Equation (2) can be used to estimate the average treatment effect of high-intensity caregiving. Specifically, the outcome variable of the estimating equation is *MH*_*i,t*_ (i.e., the mental health of informal caregivers) and a treatment *HI*_*i,t*_ (i.e., a measure of high-intensity caregiving). A dummy variable indicating the treatment condition *HI*_*i,t*_ (i.e., *HI*_*i,t*_ = 1 if informal caregiver *i* engages in high-intensity caregiving at time *t*, and 0 otherwise) is directly entered into the regression equation and the outcome variable *MH*_*i,t*_ is observed for both *HI*_*i,t*_ = 1 and *HI*_*i,t*_ = 0.

$$ M{H}_{i, t}=\eta H{I}_{i, t}+{z}_{i, t}{\textstyle \hbox{'}}\omega +{\varepsilon}_{i, t}, $$

2 $$ H{I}_{i, t}^{*}={k}_{i, t}\hbox{'}\alpha + {u}_{i, t}, H{I}_{i, t} = 1\ \mathrm{if}\  H{I}_{i, t}^{*} > 0,\ \mathrm{and}\  H{I}_{i, t} = 0\ \mathrm{otherwise} $$

$$ \mathrm{E}\left({\varepsilon}_{i, t}\Big|{v}_{i, t}\right) = {\psi}_1{v}_{i, t},\ \mathrm{E}\left({u}_{i, t}\Big|{v}_{i, t}\right) = {\psi}_2{v}_{i, t} $$

where *k*_*i,t*_ are the covariates used to model treatment assignment. The covariates *z*_*i,t*_ and *k*_*i,t*_ are unrelated to the error terms.

According to Wooldridge’s procedure [28], the control function approach can be used to estimate the following model for a binary treatment variable. After obtaining the generalized residuals (*grHI*_*i,t*_) of *HI*_*i,t*_ in the regression equation, the control function regression is as follows:

3 $$ M{H}_{i, t}={\theta}_0+{\theta}_1 M{H}_{i, t-1}+{\theta}_2 H{I}_{i, t}+{z}_{i, t}{\textstyle \hbox{'}}{\theta}_3+{\theta}_4 g r H{I}_{i, t}+{\theta}_5\left( H{I}_{i, t}\times grH{I}_{i, t}\right)+{\varepsilon}_{i, t} $$

This second-stage regression equation (3) including the generalized residual is without exclusion restrictions due to the residuals’ nonlinearity [29].^23^ Estimating Eq. (3) allows researcher to analyze whether high-intensity caregiving is associated with worse mental health among informal caregivers. Note that because the effect of moderate or serious mental health was considered persistent for informal caregivers, the lagged mental health variable was used.

Although there are no required exclusion restrictions for endogenous selection models [29, 30], such restrictions are useful for ensuring appropriate identification of the parameters. To control for any factors that might influence the probability of high-intensity caregiving, the initial value of caregiving was included as an exclusion restriction. Notably, there were positive correlations between the initial value of caregiving and high-intensity caregiving among non-working caregivers (0.163, *p* < .01). In contrast, there were no correlations between the initial value of caregiving and serious mental distress among this same employment group. In the preliminary analysis, I observed that the initial value of caregiving had significant (*p* < .01) positive effects on the high-intensity caregiving of both non-working caregivers and irregular employees.

## Results and Discussion

### Descriptive statistics

Table 1 presents the descriptive statistics of the employment status groups. The proportion of inactive persons who engaged in high-intensity caregiving (20–40 h or 40 h or more) was 27.2% (15.4%, 11.8%), which was higher than the 20.8% (12.8%, 8%) among informal caregivers with part-time work. Of all workers, the proportion of workers taking family care leave who engaged in high-intensity caregiving was the highest among the employment status groups, at 50.3% (22.8%, 27.4%). Regarding the outcomes of interest, the rate of moderate mental distress among inactive persons was 31.6%, which was slightly higher than the 31.1% for informal caregivers with part-time work. In contrast, workers who were taking family care leave had a higher prevalence of severe mental distress (14.3%) than did inactive persons (11.1%).

**Table 1:** Descriptive statistics of four samples by employment status

Variables	Non-working									Irregular employees			Regular employees		
	Total			Inactive persons			Care leave								
	N	Mean	S.D.	N	Mean	S.D.	N	Mean	S.D.	N	Mean	S.D.	N	Mean	S.D.
Dependent variables															
High-intensity caregiving	4715	0.309	0.46	3445	0.272	0.44	784	0.503	0.50	6978	0.208	0.41	3580	0.144	0.35
High-intensity caregiving (initial)	3957	0.144	0.35	2943	0.131	0.34	618	0.244	0.43	5804	0.085	0.28	2852	0.059	0.24
High-intensity caregiving (20–40 h)	4715	0.165	0.37	3445	0.154	0.36	784	0.228	0.42	6978	0.128	0.33	3580	0.090	0.29
Ultra-high-intensity caregiving (40 h or more)	4715	0.145	0.35	3445	0.118	0.32	784	0.274	0.45	6978	0.080	0.27	3580	0.054	0.23
Sum of K6	4483	4.834	4.71	3277	4.493	4.57	750	5.991	4.97	6540	4.272	4.47	3440	3.903	4.29
Serious mental health (13 ≤ K6)	4715	0.119	0.32	3445	0.111	0.31	784	0.143	0.35	6978	0.115	0.32	3580	0.082	0.27
Moderate mental health (5 ≤ K6 < 13)	4715	0.344	0.47	3445	0.316	0.47	784	0.430	0.50	6978	0.311	0.46	3580	0.297	0.46
Explanatory variables															
Co-residential caregiving	4715	0.743	0.44	3445	0.721	0.45	784	0.809	0.39	6978	0.791	0.41	3580	0.825	0.38
Sex (male = 1)	4715	0.145	0.35	3445	0.121	0.33	784	0.121	0.33	6978	0.353	0.48	3580	0.638	0.48
Age	4715	57.56	2.97	3445	57.87	2.91	784	56.72	2.93	6978	57.11	2.99	3580	56.07	2.71
Married	4715	0.737	0.44	3445	0.732	0.44	784	0.754	0.43	6978	0.765	0.42	3580	0.792	0.41
Divorced or widowed	4715	0.010	0.10	3445	0.008	0.09	784	0.010	0.10	6978	0.013	0.11	3580	0.016	0.13
Care recipient															
Father	4715	0.144	0.35	3445	0.139	0.35	784	0.163	0.37	6978	0.173	0.38	3580	0.221	0.41
Mother	4715	0.478	0.50	3445	0.472	0.50	784	0.469	0.50	6978	0.472	0.50	3580	0.532	0.50
Father-in-law	4715	0.083	0.28	3445	0.077	0.27	784	0.106	0.31	6978	0.094	0.29	3580	0.077	0.27
Mother-in-law	4715	0.287	0.45	3445	0.298	0.46	784	0.283	0.45	6978	0.279	0.45	3580	0.211	0.41
Caregiver’s income source, health status															
Relative resources or logged wage rate	4715	1.627	2.09	3445	1.712	2.10	784	1.638	2.05	5356	0.818	0.28	2985	0.927	0.15
Dummy for difficulty in daily life activities	4557	0.175	0.38	3339	0.181	0.39	752	0.156	0.36	6690	0.116	0.32	3477	0.078	0.27
Dummy for medication or doctor’s consultation	4715	0.297	0.46	3445	0.317	0.47	784	0.224	0.42	6978	0.289	0.45	3580	0.304	0.46
Dummy for hospitalization during the past year	4715	0.024	0.15	3445	0.027	0.16	784	0.011	0.11	6978	0.016	0.13	3580	0.018	0.13
Diabetes	4024	0.093	0.29	2970	0.095	0.29	659	0.064	0.24	5831	0.091	0.29	3183	0.094	0.29
Heart disease (angina, myocardial infarction)	4018	0.041	0.20	2962	0.044	0.20	661	0.024	0.15	5825	0.042	0.20	3183	0.038	0.19
Cerebral stroke	4017	0.022	0.15	2961	0.024	0.15	660	0.006	0.08	5822	0.015	0.12	3180	0.013	0.11
Hypertension	4034	0.234	0.42	2972	0.248	0.43	665	0.203	0.40	5855	0.263	0.44	3195	0.250	0.43
Hyperlipidemia	4026	0.166	0.37	2968	0.175	0.38	661	0.127	0.33	5837	0.163	0.37	3192	0.196	0.40
Cancer	4012	0.032	0.18	2958	0.038	0.19	659	0.009	0.10	5808	0.021	0.14	3173	0.020	0.14
Caregiver’s lifestyle															
Having friends/acquaintances	4642	0.829	0.38	3390	0.833	0.37	776	0.825	0.38	6789	0.867	0.34	3527	0.841	0.37
Dummy for current smoker	4715	0.136	0.34	3445	0.118	0.32	784	0.125	0.33	6978	0.226	0.42	3580	0.295	0.46
Dummy for almost every day or every day drinker	4715	0.303	0.46	3445	0.299	0.46	784	0.328	0.47	6978	0.289	0.45	3580	0.258	0.44
Dummy for regular physical activity	4715	0.542	0.50	3445	0.552	0.50	784	0.503	0.50	6978	0.420	0.49	3580	0.426	0.49

High-intensity caregiving (initial) refers to high-intensity caregiving in 2005, when the LSMEP launched

Sources: Longitudinal Survey of Middle-aged and Elderly Persons 2005, 2006, 2007, 2008 and 2009

Most workers taking family care leave were female, and the proportion of these workers who had a drinking habit was the largest among all informal caregivers studied. If there were a simultaneity issue for non-working female caregivers, the relationship between serious mental distress and risky health behavior such as a drinking habit or a smoking habit would likely be positive even after controlling for the other covariates. Considering this possible simultaneity, I did not use lifestyle variables as covariates of the high-intensity caregiving function in further analyses.

As noted above, I constructed a binary variable of co-residential caregiving for each family member type other than spouse. A change from 1 to 0 in this variable indicated a shift from co-residential caregiving to non-residential caregiving.^24^ The rate of co-residential caregiving was lowest (0.721) among inactive caregivers, while that of regular employees was 0.825.

Around 7% of inactive persons did not answer the question about their hours of caregiving per week. As such, they were excluded from the remainder of the analyses.

#### Determinants of high-intensity caregiving

To estimate the determinants of high-intensity caregiving, I estimated random-effects probit models for regular employees, irregular employees, and non-working caregivers. The estimation results for both non-working caregivers and irregular employees showed that co-residential caregiving was significantly positively related to high-intensity caregiving. In contrast, co-residential caregiving had no significant effect on high-intensity caregiving among regular employees. Thus, I focused only on the determinants of high-intensity caregiving among the non-working informal caregivers and irregular employees.^25^

Because there are no available means of dealing with caregiving histories with the initial year missing, left-censored spells are typically omitted from this analysis. To manage the left-censoring problem in this study, I took into account the initial period choice, because state dependence implies that initial period choices depend endogenously on earlier choices causing left censoring. Thus, I included the initial value of high-intensity caregiving as an explanatory variable in the regression models.

Table 2 shows the estimated determinants of high-intensity caregiving among non-working informal caregivers and irregular employees. Among both groups, past high-intensity caregiving (20–40 h and 40 h or more) and the initial value of high-intensity caregiving had significant (*p* < .01) positive effects on current high-intensity caregiving. Notably, the coefficients for high-intensity caregiving of 20–40 h were larger than were those of ultra-high-intensity caregiving (i.e., 40 h or more); this indicated that caregivers who experienced high-intensity caregiving 20–40 h more likely to continue that high-intensity caregiving than were caregivers who had experienced ultra-high-intensity caregiving. Considering the non-proportional relationship between hours of caregiving and caregivers’ burden found by Arai and Ueda (2003), the perceived burden of caregivers who experienced high-intensity caregiving (20–40 h) might be the heaviest among all informal caregivers. This finding was considered in the specification of the continuation of caregiving function described below. It should be noted that being male had a marginally significant negative effect on the likelihood of high-intensity caregiving at the 10% significance level.

**Table 2:** High-intensity caregiving functions

Independent variables	Non-working	Irregular employees	Regular employees
High-intensity caregiving (−1)			
20–40 h (−1)	0.919***	1.031***	1.013***
	(0.116)	(0.136)	(0.199)
40 h or more (−1)	0.744***	0.538***	0.374*
	(0.104)	(0.116)	(0.194)
High-intensity caregiving (initial)	0.573***	0.550***	0.579***
	(0.115)	(0.134)	(0.189)
m (non-attendance of health checkup)	8.613***	2.081	0.259
	(2.795)	(2.727)	(3.661)
Care leave	0.521***		
	(0.0835)		
Relative resources squared	0.0131**		
	(0.00606)		
Relative resources/wage rate	−0.0538**	−0.134	0.517*
	(0.0222)	(0.126)	(0.275)
Co-residential caregiving	0.285***	0.0753	0.0641
	(0.0753)	(0.0802)	(0.108)
Sex (male = 1)	−0.199*	−0.135*	−0.401***
	(0.106)	(0.0796)	(0.100)
Care recipients			
Father	−0.0517	−0.0366	0.129
	(0.0954)	(0.0935)	(0.107)
Mother	0.137*	0.0564	−0.0185
	(0.0770)	(0.0789)	(0.0988)
Father-in-law	0.274**	−0.0701	0.287*
	(0.121)	(0.116)	(0.151)
Mother-in-law	0.0332	0.0776	−0.162
	(0.0838)	(0.0874)	(0.119)
Health status and Medical care use			
Medication or doctor’s consultation	0.243**	0.0613	0.0386
	(0.107)	(0.105)	(0.127)
Hospitalization during the past year	−0.577**	0.263	−0.649*
	(0.233)	(0.247)	(0.390)
Difficulty in daily life activities	−0.00896	0.0340	−0.289*
	(0.0863)	(0.103)	(0.171)
Hypertension	−0.201*	−0.0996	0.00882
	(0.104)	(0.100)	(0.122)
Hyperlipidemia	−0.182**	−0.118	0.00838
	(0.0906)	(0.0903)	(0.104)
Constant	−4.304***	−3.358***	−1.365
	(1.160)	(1.133)	(1.502)
lnσ_u_^2^	−1.266***	−0.716***	−1.815**
	(0.384)	(0.274)	(0.913)
σ_u_	0.531***	0.699***	0.403**
	(0.101)	(0.095)	(0.184)
Intra-class correlation (σ_u_^2^/(1 + σ_u_^2^))	0.220	0.328	0.140
Likelihood-ratio test of ρ = 0 [chi^2^(1)]	11.32	28.96	1.62
Prob ≥ chi^2^	0.00	0.00	0.10
Log likelihood	−1501.54	−1610.13	−733.07
*N*	2879	3580	2063

High-intensity caregiving (initial) refers to high-intensity caregiving in 2005. m(X) means time-average of time-variant explanatory variable (X)

Age, educational attainment, marital status, residence, having cancer, cerebral stroke, diabetes, heart disease, and friends were included as covariates

Standard errors in parentheses. *** *p* < 0.01, ** *p* < 0.05, * *p* < 0.1

Among non-working informal caregivers, taking care leave, co-residential caregiving, medication or doctor’s consultation, and caregiving for a father-in-law had positive effects on high-intensity caregiving. Furthermore, both relative resources and the squared term of relative resources were significantly related to high-intensity caregiving (*p* < .05). Relative resources—namely, the difference between logged spouse’s income and logged respondent’s income—had a value of 0 if the respondent was single. The quadratic function of relative resources had a minimum value of −0.055 at 2.05, which would mean that there is a high likelihood of high-intensity caregiving among non-working caregivers with a large difference between the logged spouse’s income and logged respondent’s income.

The relative resources squared (*rrs*) is considered a proxy variable of the shadow price of informal care for non-working caregivers (inactive or taking care leave) because individuals with higher income tend to spend less hours in informal caregiving. Therefore, the *rrs* includes the opportunity costs of informal caregivers, which shows the monetary value of the alternative use of hours of caregiving as market work or leisure time. The Pearson correlation between *rrs* and household income ratio (*hir*) is 0.501, and *hir* was not statistically significant at the 10% level when using *rrs* and *hir* as explanatory variables of the high-intensity caregiving function, suggesting that multicollinearity may exist between them. The *hir* is the ratio of household income to the poverty line. The poverty line, for example 1.25 million yen in 2009, was obtained from the Comprehensive Survey of Living Conditions. The *rrs* was associated with both the household income status and socioeconomic status, although the definition of socioeconomic status is an arbitrary one.^26^ Non-working caregivers with less education were not likely to be accepted in the labor force, and tended to engage in high-intensity caregiving in their household. The estimation results of random-effects models of *rrs* are shown in the Appendix. The Hausman tests supported the random-effects models.

In contrast, having hyperlipidemia and being hospitalized during the past year had negative effects on high-intensity caregiving (*p* < .05). Being married also had negative effects on high-intensity caregiving (see Appendix), suggesting that the “never married” group (i.e., the comparison group) were more likely to engage in high-intensity caregiving. The proportion of inactive persons who were never married was 0.26.

I determined the intra-class correlation coefficients (ICCs) from an error-components panel data model using the equation $\mathrm{I}\mathrm{C}\mathrm{C}=\kern0.5em {\sigma}_u^2/\left(1+{\sigma}_u^2\right)$, where ${\sigma}_u^2$ represents the variance of the unobserved individual effect. ICCs are used to measure the proportion of the total unexplained variation that can be attributed to individual effects. Here, the ICC represents the correlations between high-intensity caregiving across the different periods of observation; an ICC value close to unity indicates a high persistence of high-intensity caregiving.

The estimation results of the DREP model for irregular employees showed that unobserved heterogeneity was a strong source of persistence in high-intensity caregiving; specifically, unobserved heterogeneity accounted for 32.8% of the unexplained variance in high-intensity caregiving. Considering the population distribution of unobserved heterogeneity, I obtained population-averaged parameters as ${\beta}_a=\beta /\sqrt{\left(1+{\sigma}_u^2\right)}$ . The averaged parameter of lagged high-intensity caregiving (20–40 h) among irregular employees was 0.845 (1.031/1.220), while that among non-working caregivers was 0.812 (0.919/1.132). The averaged parameters of lagged high-intensity caregiving (40 h or more) were relatively smaller, at 0.441 among irregular employees and 0.657 among non-working caregivers. The degree of state dependence between previous and current high-intensity caregiving (20–40 h) exhibited moderate persistence.

The averaged parameter of co-residential caregiving among non-working caregivers was 0.285; in other words, almost 25% of the persistence of high-intensity caregiving was increased by co-residential caregiving overall. Furthermore, almost 71% of the persistence of high-intensity caregiving was increased by co-residential caregiving for a father-in-law and having a doctor’s consultation. The time average of non-attendance of health checkup had a positive and significant (*p* < .01) effect on high-intensity caregiving. This suggests that non-working caregivers who had not received health checkups during the current period had a high likelihood of engaging in high-intensity caregiving.^27^ These tendencies were not found for irregular employees.

#### Effects of high-intensity caregiving on caregivers’ mental health

The dependent variable of the mental health function was serious mental distress as measured by the K6. The independent variables of the first-stage pooled probit model were age, care leave, co-residential caregiving, caregivers’ health status, relation of care recipients, educational attainment, having friends or acquaintances, hospitalization, marital status, medication or doctor’s consultation, residence, sex, and relative resources or wage rate. The initial value of caregiving was also included as an exclusion restriction. The estimation results of the DREP models showed that high-intensity caregiving was associated with worse mental health among non-working caregivers. Indeed, there were distinct impacts of high-intensity caregiving on mental health of informal caregivers. However, high-intensity caregiving did not have any effect on serious mental health among irregular employees.

Table 3 shows that both the generalized residual and the interaction (HI × GR) were not significant (*p* > .05), and that high-intensity caregiving was an exogenous variable of caregivers’ mental health. The averaged parameter of high-intensity caregiving was 1.086 (1.234/1.137) among non-working caregivers. This result indicates that caregivers who had engaged in high-intensity caregiving would have a current mental health status of serious mental distress when their mental health was moderate (5 ≤ K6 < 13) in the past period.^28^

**Table 3:** Caregivers’ mental health functions

	Non-working	Irregular employees
Dependent variables	Serious mental distress (13 ≤ K6)	
Independent variables		
Mental health (−1) (1 = moderate, 2 = serious)	0.498***	0.381***
	(0.0697)	(0.0609)
Mental health (initial)	0.478***	0.346***
	(0.0863)	(0.0718)
m(proxy of formal care use)	−115.3***	−49.58*
	(36.30)	(28.14)
High-intensity caregiving (HI)	1.234**	−0.208
	(0.496)	(1.148)
Generalized residual (GR)	−0.684*	0.0828
	(0.372)	(0.922)
HI × GR	0.00418	0.143
	(0.395)	(0.810)
Care leave	−0.181	
	(0.163)	
Health status		
Difficulty in daily life activities	0.487***	0.279**
	(0.108)	(0.111)
Hospitalization during the past year	0.605**	0.188
	(0.252)	(0.274)
Diabetes	0.159	0.223
	(0.156)	(0.139)
Heart disease	0.299	0.487***
	(0.200)	(0.170)
Cerebral stroke	−0.236	−0.0761
	(0.302)	(0.348)
Hypertension	−0.0266	0.160
	(0.144)	(0.118)
Hyperlipidemia	−0.0348	0.0595
	(0.135)	(0.107)
Having friends/acquaintances	−0.184	−0.360***
	(0.114)	(0.116)
Constant	1.466	0.313
	(1.503)	(1.212)
lnσ_u_^2^	−1.231**	−1.438**
	(0.531)	(0.634)
σ_u_	0.540***	0.487***
	(0.143)	(0.154)
Intra-class correlation (σ_u_^2^/(1 + σ_u_^2^))	0.226	0.192
Likelihood-ratio test of ρ = 0 [chi^2^(1)]	6.19	3.88
Prob ≥ chi^2^	0.00	0.02
Log-likelihood	−637.77	−830.43
*N*	2785	3448

Mental health (initial) refers to high-intensity caregiving in 2005. m(X) means the time-average of a time-variant explanatory variable (X)

Age, care recipients, educational attainment, marital status, medication or doctor’s consultation, residence, sex, and having cancer were included as covariates

Standard errors in parentheses. *** *p*<0.01, ** *p*<0.05, * *p*<0.1

The averaged parameter of the lagged mental health of non-working caregivers was 0.438. This indicates that the degree of state dependence between previous mental health and current serious mental distress reflected moderate persistence. All the initial values of mental health—which ranged from serious (2) to light (0)—were significantly related with current mental health at the 1% level. Depressive symptoms were considered persistent for non-working caregivers.

To investigate the hypothesis that respite care was useful in alleviating mental distress among informal caregivers who were engaged in high-intensity caregiving, a time-averaged proxy variable of formal care use was used. Among non-working caregivers, when this variable was included as a covariate of random-effects, it was significantly negatively related to mental distress (*p* < .01). This suggests that respite care was indeed useful in alleviating mental distress for non-working caregivers.

There was a high likelihood of high-intensity caregiving among non-working caregivers who had not received a health checkup during the current period. Therefore, we might consider that caregivers whose time discount rate is high do not give much thought to their future health status and therefore are more likely to engage in high-intensity caregiving. Unobservables such as higher time discount rates could have increased the rate of high-intensity caregiving, which in turn would correlate to unobservables that worsened the mental health of caregivers.

#### Effects of high-intensity caregiving on continuation of caregiving

Given the findings of the previous analysis that high-intensity caregiving was associated with worse mental health among non-working caregivers, I focused on the impact of current high-intensity caregiving on continuation of caregiving only in this group. More specifically, since the transition from non-working to part-time work was infrequent among informal caregivers, I extracted those who were not working at time *t* and did not provide high-intensity care at time *t-1* before running the regression. During the sample period, about 6% of non-working caregivers in period *t* transitioned to part-time work in the next period. In contrast, 29% of workers in period *t* transitioned to non-working caregivers in the next period.

Table 4 shows the estimation results of the continuation of caregiving functions among non-working caregivers. The dependent variable of continuation of caregiving was informal caregiving in the next period, which includes non-residential caregiving. To control for factors influencing the probability of high-intensity caregiving among non-working caregivers when analyzing continuation of caregiving, I included respondents’ answer to the question “Do you sometimes try to rest to maintain your health?” as an exclusion restriction.^29^ I observed a negative correlation between this exclusion restriction variable and high-intensity caregiving among non-working caregivers (−0.039, *p* < .05). In contrast, there were no correlations between this variable and continuation of caregiving.

**Table 4:** Continuation of caregiving of non-working caregivers

Independent variables		HI not provided in previous period
Co-residential caregiving	−0.0101	−0.0417
	(0.122)	(0.129)
Co-residential caregiving (initial)	0.456***	0.573***
	(0.104)	(0.121)
m(proxy of formal care use)	−62.58**	−68.33**
	(26.23)	(29.21)
High-intensity caregiving (HI)	1.047*	1.232**
	(0.537)	(0.570)
Generalized residual (GR)	−0.462	−0.363
	(0.422)	(0.434)
HI × GR	0.0907	−0.281
	(0.485)	(0.520)
Relative resources squared	0.00917	0.00598
	(0.00878)	(0.00931)
Relative resources	0.0229	0.0374
	(0.0327)	(0.0351)
Sex (male = 1)	−0.120	−0.104
	(0.157)	(0.167)
Care recipients		
Father	0.0599	0.0339
	(0.135)	(0.144)
Mother	0.408***	0.469***
	(0.111)	(0.122)
Father-in-law	−0.143	−0.117
	(0.166)	(0.178)
Mother-in-law	0.284**	0.317**
	(0.118)	(0.129)
Health status		
Difficulty in daily life activities	−0.0223	−0.0518
	(0.124)	(0.140)
Diabetes	0.0400	0.0541
	(0.167)	(0.184)
Heart disease	−0.168	−0.0844
	(0.225)	(0.242)
Cerebral stroke	−0.0348	0.0627
	(0.327)	(0.340)
Hypertension	0.216	0.231
	(0.152)	(0.164)
Hyperlipidemia	0.0545	−0.0108
	(0.123)	(0.132)
Having friend	0.206*	0.274**
	(0.124)	(0.134)
Constant	1.492	0.926
	(1.330)	(1.445)
lnσ_u_^2^	−0.422	−0.376
σ_u_	0.809***	0.828***
	(0.141)	(0.157)
Intra-class correlation (σ_u_^2^/(1 + σ_u_^2^))	0.396	0.407
Likelihood-ratio test of ρ = 0 [chi^2^(1)]	18.10	15.70
Prob ≥ chi^2^	0.00	0.00
Log-likelihood	−1005.92	−857.71
*N*	1743	1454

Co-residential caregiving (initial) refers to high-intensity caregiving in 2005. Age, educational attainment, hospitalization, marital status, medication or doctor’s consultation, residence, and having cancer were included as covariates

Standard errors in parentheses. *** *p*<0.01, ** *p*<0.05, * *p*<0.1

The second-stage estimation results of the DREP models showed that current high-intensity caregiving was significantly associated with continuation of caregiving (*p* < .05). Because the averaged parameter of high-intensity caregiving was 0.949 (1.232/1.299) when high-intensity caregiving was not provided during the previous period, caregiving was considered persistent for non-working caregivers. Notably, there was a high likelihood of continuation of caregiving when the care recipient was a mother or mother-in-law, regardless of the provision of high-intensity caregiving during the previous period. However, non-working caregivers did not tend to continue high-intensity caregiving for more than three years because current high-intensity caregiving was not associated with the continuation of caregiving when high-intensity caregiving provided during the previous period was included in the regression. The time-averaged proxy variable of formal care use was significantly and negatively related to continuation of caregiving, indicating that current respite care has a negative effect on continuation of informal caregiving. This suggests that caregivers might elect to use formal care when formal care services are readily available in the next period.

## Conclusions

The results of the present study offered robust support for a causal relationship between high-intensity caregiving and mental health problems. This suggests that supporting family caregivers is an important public health issue. While little was known until now about the specifics of the negative relation between high-intensity caregiving and caregivers’ mental health in Japan, the present study shed some light on this area.

Three major findings were uncovered: First, hours of caregiving is thought to influence the continuation of high-intensity caregiving among non-working informal caregivers and irregular employees. Specifically, caregivers who experienced high-intensity caregiving (20–40 h) tended to continue with it to a greater degree than did caregivers who experienced ultra-high-intensity caregiving (40 h or more). Second, there were distinct impacts of high-intensity caregiving on the mental health of informal caregivers. High-intensity caregiving was associated with worse mental health among non-working caregivers, but had no effect on the serious mental distress of irregular employees. Caregivers who had engaged in high-intensity caregiving tended to exhibit serious mental distress currently when they exhibited mental health in the previous period. Furthermore, the time-averaged proxy variable of formal care use was significantly negatively related to mental distress; this suggests that respite care was useful for non-working caregivers. Finally, non-working caregivers did not tend to continue high-intensity caregiving for more than three years, regardless of co-residential caregiving. This is because current high-intensity caregiving was not associated with the continuation of caregiving when I included high-intensity caregiving provided during the previous period in the regression. In sum, caregiving tends to persist among non-working caregivers who experienced high-intensity caregiving for a year. Thus, supporting non-working intensive caregivers should be a priority public health issue.

Although a shift from caregiving to employment has been observed when formal care services are readily available, due to the limited data, I did not fully control for simultaneity between informal caregiving, the eligibility levels of care recipients, and formal care use. This simultaneity problem should be examined in further analyses.

## Appendix

**Table 5:** High-intensity caregiving functions (continuation of Table 2)

Independent variables	Non-working	Irregular employees	Regular employees
Age and Marital status			
Age	0.0187	0.0269**	−0.006
	(0.0129)	(0.0126)	(0.0169)
Married	−0.349***	0.0361	−0.0957
	(0.101)	(0.104)	(0.148)
Educational attainment			
Junior high school	−0.00893	−0.101	0.0893
	(0.106)	(0.108)	(0.151)
Vocational school	−0.0298	0.190*	−0.0753
	(0.111)	(0.114)	(0.150)
Junior college or technical college	0.121	0.0536	−0.120
	(0.0921)	(0.107)	(0.156)
University	−0.0657	−0.0973	−0.157
	(0.106)	(0.0986)	(0.105)
Graduate school	−0.145	0.000837	0.284
	(0.513)	(0.388)	(0.302)
Residence			
Rental housing	0.0491	0.227*	0.197
	(0.137)	(0.121)	(0.163)
Company residence	−0.371	0.560	0.254
	(0.522)	(0.425)	(0.450)
Other residences	−0.169	0.357	0.510
	(0.263)	(0.225)	(0.335)
Health status and Medical care use			
Diabetes	−0.0372	−0.151	−0.0755
	(0.122)	(0.125)	(0.148)
Heart disease	0.0851	0.0306	0.320*
	(0.166)	(0.167)	(0.187)
Cerebral stroke	0.0890	0.203	−0.681
	(0.222)	(0.259)	(0.545)
Cancer	−0.0948	−0.00433	0.435
	(0.212)	(0.238)	(0.300)
Having friend/acquaintances	−0.0823	0.0586	0.121
	(0.0867)	(0.103)	(0.122)

Divorced or widowed was dropped because of collinearity

Standard errors in parentheses. *** *p* < 0.01, ** *p* < 0.05, * *p* < 0.1

**Table 6:** Relative resources squared and household income status

	Random-effects model		Fixed-effects model	
Dependent variable	Relative resources squared			
Independent variables	[1]	[2]	[3]	[4]
Low socioeconomic status	−0.561**	−0.447*	−1.855**	−1.855**
	(0.241)	(0.250)	(0.752)	(0.752)
High socioeconomic status		0.744*		0.147
		(0.423)		(0.705)
Household income ratio	0.392***	0.390***	0.311***	0.311***
	(0.0108)	(0.0109)	(0.0125)	(0.0125)
Sending living expenses for non-housemates	0.886***	0.859***	−0.0298	−0.0327
	(0.256)	(0.256)	(0.399)	(0.399)
Constant	6.288***	6.179***	7.560***	7.549***
	(0.188)	(0.198)	(0.405)	(0.409)
R-squared	0.255	0.256	0.243	0.243
Hausman test				
chi^2^ (Prob > chi^2^)	[1] vs [3] 182.10 (0.00)		[2] vs [4] 179.95 (0.00)	
*N*	4097	4097	4097	4097

Standard errors in parentheses. *** *p* < 0.01, ** *p* < 0.05, * *p* < 0.1

## Abbreviations

DREP model: Dynamic random-effects probit model
K6: Kessler 6 non-specific distress scale
LSMEP: Longitudinal Survey of Middle-aged and Elderly Persons
LTCI: Long-term care insurance
MHLW: Ministry of Health, Labor and Welfare

## Footnotes

1 Hirst specifically examined changes in experienced distress levels between caregivers and non-caregivers surrounding transitions into and out of caregiving [2].

2 Intensive caregivers tend to have lower incomes compared to non-intensive caregivers. Notably, intensive care is predominately provided by the spouse of the care recipient, and most intensive caregivers are 50–64 years old [1].

3 The Japanese government introduced a community-based, prevention-oriented long-term care (LTC) benefit targeted at low-care-needs seniors in 2006. Since April 2006, there are seven levels of care needs certification under public LTCI: the two lowest levels are “assistance required” (*yo-shien* in Japanese) and the remaining five levels refer to “care required” (*yo-kaigo* in Japanese). Similarly, in 2008, Germany introduced “carrot-and-stick” financial incentives for sickness funds that proved successful at rehabilitating and moving LTC users from institutions to lower-care settings [1].

4 In Japan, spending on public LTC increased 40% over the six years from 2006 to 2012. A further increase in LTC expenses is expected as the Japanese population continues to age.

5 In Japan, respite care, or short-stay care (i.e., having the care recipient spend a few nights at a time in a nursing home) is widely used (350,000 stays per month), but the number of institutional beds is inadequate because of high demand [31].

6 Female caregivers, who were expected to have homemaking skills according to family-bound gender roles, were the least likely to use formal visiting homecare services [6].

7 According to [1], across the Organization for Economic Cooperation and Development (OECD) member countries, more than one in ten adults over 50 years of age provide (usually) unpaid help with personal care to people with functional limitations. Close to two-thirds of such caregivers are women.

8 As they suggested, the objective burden of informal care does not include how caregivers experience their caregiving tasks.

9 Because long-term care is time consuming, the flexibility of non-regular work might be preferable for informal caregivers [16].

10 In contrast, informal care might serve as a complement to formal care that cannot be replaced by family or other informal caregivers, such as outpatient care requiring professional practice.

11 First, 2,515 districts were randomly selected from the 5,280 districts used in the MHLW’s nationwide, population-based “Comprehensive Survey of the Living Conditions of People on Health and Welfare,” conducted in 2004. These 5,280 districts had been randomly selected from about 940,000 national census districts. Second, 40,877 residents aged 50–59 years as of October 30, 2005 were randomly selected from each selected district proportionate to its population size. A total of 34,240 individuals responded to the first survey wave (response rate: 83.8%), while 32,285 subjects returned the questionnaires for the second wave (response rate: 92.2%). The third to fifth waves of the survey were conducted in 2007–2009 and consisted of 30,730 (response rate: 95.4%), 29,605 (96.2%), and 28,736 (97.3%) respondents, respectively. No new respondents were added after the first wave.

12 Socially disadvantaged families may be more likely to engage in caregiving and have fewer labor market opportunities [1].

13 Prochaska et al. [32] identified a K6 ≥ 5 as the optimal lower threshold cut-off for moderate mental distress according to a receiver operating characteristic curve. Prochaska et al. also found that mental distress was more prevalent among those with lower education levels, those who were unemployed and looking for work, those who were not married, binge drinkers and current and former smokers, those who were not regularly physically active, and those who were obese.

14 Regarding the hours of informal caregiving in 2007 (2010), the proportion of main caregivers who provided “2 h or more per day” for care recipients with a care level of 3 was 0.577 (0.663), while the proportion who provided “almost all day” was 0.309 (0.338) [Comprehensive Survey of the Living Conditions of People on Health and Welfare, 2007, 2010].

15 This tendency was the same for caregivers who were not co-residing with the care recipient.

16 Co-residential caregiving is also likely to involve considerable transition costs, such that caregivers are more likely to remain caregivers in the future [33].

17 In 2010, 41% of co-residential females in their 50s or 60s were the primary caregivers according to the Comprehensive Survey of Living Conditions 2010 by the MHLW (Available from http://www.mhlw.go.jp/toukei/saikin/hw/k-tyosa/k-tyosa10/4-3.html) (in Japanese).

18 Although family caregivers could rely only on home help, day care, and other home and community-based services, this is not enough to relieve their burden so long as caring is regarded as a full-time duty [31]. Furthermore, based on a survey of 2,530 family caregivers, Suzuki et al. found that there is insufficient provision of short-term stays, day services, and home-helper services [5].

19 Assuming that initial conditions are exogenous, the random effects variance is restricted to zero. This indicates that there is no unobserved heterogeneity in participation probabilities.

20 Higher income was found to be positively related to latent health stock, while low educational attainment, difficulty in daily life activities, and care for family members were negatively related [27]. They used health stock as a dummy variable, which took on the value of 1 if the latent health stock was good and 0 otherwise.

21 The discrete-factor random-effects estimator approach provides econometricians with an easy method of jointly estimating two (or more) behaviors of interest [34].

22 This assumption enables a specific correlation structure between unobservables that affects the treatment and the unobservables that affect potential outcomes.

23 Vella proposed that a generalized residual has two important characteristics: it has a mean zero over the whole sample, and it is uncorrelated with the explanatory variables in the first-step probit model [29].

24 For informal caregiving overall, a shift from 1 to 0 indicated a shift from informal caregiving to no longer being an informal caregiver (i.e., beginning use of formal care use or the death of the care recipient).

25 It should be noted here that 575 of the inactive persons changed from previous non-working status to current working status, while 263 changed from previous working status to current non-working status. However, the dataset is weakly balanced because each panel contains the same number of observations but not the same time points.

26 Because the *rrs* is distributed in a biased manner among non-working caregivers, the explanation of the *rrs* depends on *hir*, sending living expenses for non-housemates (*slc*), and high or low socioeconomic status (*hses* or *lses*). The coefficient of *lses* was −0.56, and its magnitude was about 1.43 times that of *hir*. When censoring the caregivers whose *rrs* was zero (*N* = 1460), the coefficient of *lses* of the random-effects tobit model was −0.99, and its magnitude was about 2.28 times that of *hir* (not shown in Table 6). About half of the informal caregivers belonged to the *lses.* The *lses* includes non-married, non-working caregivers whose educational attainment is low (less than high school). The *hses* consists of non-working caregivers who are married and whose educational attainment is high (university level or above).

27 Using a subset of non-working informal caregivers who had received health checkups before a change in medication, I examined whether a change in medication or a doctor’s consultation had a negative effect on health checkup attendance. No such effect was found (*p* > .05)*.* I also confirmed that high-intensity caregiving had no negative effect on non-working informal caregivers’ health checkup attendance (*p* > .05).

28 The generalized residual was significant at the 5% level when including “unemployed” in the non-working group; thus, it may be that poor mental health due to unemployment can cause people to choose to stay in home and provide informal care.

29 The independent variables of the first-stage pooled probit model were age, care leave, co-residential caregiving, caregivers’ health status, care recipients, educational attainment, having friends or acquaintances, hospitalization, marital status, medication or doctor’s consultation, residence, sex, relative resources or wage rate, and the exclusion restriction.
